# Cross-cultural adaptation and validity of the Chinese version of the Oxford elbow score

**DOI:** 10.1186/s13018-020-02100-y

**Published:** 2020-11-26

**Authors:** James Reeves Mbori Ngwayi, Jie Tan, Ning Liang, Emmanuel Gildas Eric Sita, Daniel Edward Porter

**Affiliations:** 1 School of Clinical Medicine, Tsinghua University, Beijing, 100084 China; 2 School of Clinical Medicine, Fujian Medical University, Fujian, 350122 China; 3 Department of Orthopedics, Beijing Huaxin Hospital, Clinical Medicine School, Tsinghua University, Beijing, 100016 China

**Keywords:** Oxford elbow score, QuickDASH, SF-36, Elbow disorder, Reliability, Validity

## Abstract

**Background:**

The Oxford Elbow score (OES) is a patient-reported outcome measure designed to evaluate patients before and after elbow surgery. Although various translated versions of the score are available, there is no Chinese mandarin version. The aim of this study was to develop a Chinese language version of the OES and evaluate its psychometric properties for clinical use.

**Methods:**

The English version of the OES was forward translated into Chinese, followed by a backward translation into English. Then a final Chinese version was produced following expert committee discussions and pilot study of 11 patients. A smart device compatible electronic version of the OES was designed and completed by 70 patients with elbow pathology alongside the Quick-Dash and the SF-36. Reliability was assessed by measuring intraclass correlation coefficient (ICC) for test-retest reliability and Cronbach’s alpha for internal consistency. Spearman’s correlation coefficient was used to test the construct validity. Confirmatory factor analysis (CFA) was performed to evaluate the 3-factor structure of the OES.

**Results:**

The overall Cronbach’s α coefficient was 0.906 and for the 3 different domains Function, Pain, and Social-psychological was 0.806, 0.796, and 0.776 respectively. The overall intraclass correlation coefficient was 0.764 and for the three different domains Function, Pain, and Social-psychological was 0.764, 0.624, and 0.590 respectively. The Spearman’s coefficient for correlation, between the QuickDASH and OES domains Function, Pain, and Social-psychological, was − 0.824, − 0.734, and − 0.622 respectively, showing strong correlation (*r* > 0.5; *p* < 0.01). There were moderate correlations between OES domains and the physical functioning, role physical, and strong correlations with bodily pain subscales of the PCS domain of the SF-36; results were insignificant for all other subscales.

**Conclusion:**

Our translated Chinese mandarin OES version (mainland) was reliable and valid, suitable for evaluating elbow disorders in the Chinese population. Reliability was measured using both the Cronbach’s α for internal consistency and the intraclass correlation. Results were classified as “excellent” and were similar to results from the original OES. Electronic PROMs were used instead of the traditional paper-based PROMs for collection of data which was well tolerated by patients.

## Introduction

Patient-reported outcome measures (PROMs) are subjective, patient-completed questionnaires reflecting their health status and health related quality of life [1].

Most of the PROMs in use were designed originally in English. Before being used in another cultural setting, they have to undergo rigorous translation and transcultural adaptation [2].

The use of PROMs is applicable in various sectors including research, insurance, and clinical and health service evaluation by regulatory bodies [3, 4]. In the managed healthcare sector, there has been an explosion in the use of PROMs in recent years, as authorities demand that patients become more involved in decisions concerning their health welfare [5].

In the field of orthopedics and rheumatology, specific and general PROMs exist for a wide range of musculoskeletal conditions and diseases [6]. A variety of instruments have been developed and documented to asses function status and pain for elbow disorders, both objective and subjective [7].

The Oxford Elbow Score (OES) was identified as having the highest quality methodology in development in a study by The B et al. based on the Consensus-Based Standards for the Selection of Health Measurement Instruments (COSMIN) evaluation protocol [8]. Studies by Jonathan et al. identified four scores as being High-Performing Instruments including quick Disabilities of the Arm Shoulder and Hand score (QuickDASH), DASH, Oxford Elbow Score (OES), and Patient-Rated Tennis Elbow Evaluation (PRTEE) for use in patients with elbow tendinopathy [9].

The OES is a 12-item questionnaire designed for use as an outcome measure of elbow surgery. It encompasses three domains including “elbow function,” “pain,” and “social-psychological,” with each domain comprising four items. Each item has five response options scored 0 to 4, with 0 representing greater severity [10].

The OES has been translated from English into a variety of languages including French, Spanish, Danish, Finnish, German, Polish Portuguese, Swedish, Turkish, Welsh, and Dutch (https://innovation.ox.ac.uk/outcome-measures). Presently, there is no validated Chinese version of the OES; therefore, this study was aimed at developing a cross culturally adapted Chinese mandarin OES version and assessing its validity and reliability in patients with elbow disorders.

## Materials and methods

The cross-cultural adaptation of the OES was performed strictly according to the stipulated guidelines for cross-cultural adaptation of self-completed questionnaires [11].

Prior to the translation process, permission and license for the use of the OES was granted by Oxford University Innovation Limited in May 2018.

Three forward translations of the OES to Chinese were completed by three independent translators including two bilingual orthopedic surgeons and one professional translator experienced in musculoskeletal terminology. There was disparity in the forward translations regarding questions 1: “lifting things”; question 2: “carrying bags of shopping”; question 5: “controlling your life”; and question 7: “troubled by pain from elbow in bed at night.” The forward translations were reviewed by a committee of four including three bilingual orthopedic surgeons and one professional translator. The disparities were addressed and a single reconciled forward translation was adopted. The reconciled single forward version was then back translated into English. This was performed by three bilingual mother tongue translators blind to the original score, obtaining three different versions. The backward translations were compared against the original English version using the OES Concept Elaboration Report provided by Oxford Innovation. An expert committee of five (comprising three bilingual orthopedic surgeons and two professional translators) reviewed and established a prefinal OES version.

A pilot study was carried out from February–March 2019 at a general orthopedic outpatient clinic and arthroplasty specialty clinic of a level 3 general hospital in Beijing, China, involving 11 consecutive patients diagnosed with elbow pathology (four males, seven females) with an average age of 54.6 years (SD 11.9). During this pilot phase, patients were tested on their understanding and interpretation of the various questions. Patients were asked to read out and complete the form; they were asked to identify any difficult words, phrases, and ambiguities. All 11 participants confirmed understanding of the questions and therefore no further modifications were made during the final proof-reading. The Final OES version was submitted to Oxford University Innovation Ltd. and confirmed as acceptable for validity and reliability evaluation studies.

### Patients

This study was approved by the Clinical Research Ethics Committee of our institution, and all patients consented to participate in the study.

Patient inclusion criteria into the study were (1) elbow disorders which reflected those found in the original OES design paper [10] including trauma, fractures, medial and lateral epicondylitis, bursitis, posttraumatic osteoarthritis, and ulnar neuritis; (2) able to read and write Chinese; and (3) availability and usage of WeChat® app software for smart devices.

Seventy patients took part in the study (39 male, 31 female). Elbow disorders included 55 patients with epicondylitis, nine patients with elbow fractures, two patients with post-traumatic osteoarthritis, and four patients with ulnar neuritis (Table 1). Most patients were recruited consecutively from March to October 2019 at the outpatient clinic in which the previous pilot study was conducted. Several patients with fractures around the elbow during 2017–2019 were recruited by telephone follow-up.

**Table 1:** Patient demographics

Characteristics	
Gender	Number (%)
Male	39 (55.7)
Female	31 (44.3)
Diagnosis	Number (%)
Elbow Fractures	9 (12.9%)
Epicondylitis	55 (78.6%)
Osteoarthritis	2 (2.9%)
Ulnar neuritis	4 (5.7%)
	Mean (SD)
Mean age–years (SD)	44.6 (14.7)

*SD* standard deviation

The sample size of 70 was considered adequate as it fulfilled the assumption whereby the number of respondents should exceed the number of items (12) on the questionnaire by at least a factor of three [12].

In this study, only electronic versions of PROMs were used; the process was entirely paper-free. Patients downloaded the forms via *WeChat*® social media “app” by scanning a QR-code via their cell-phones after their clinic consultation. All patients received guidance on how to complete and submit the forms; they completed the OES in the outpatient clinic while the QuickDASH and SF-36 forms were sent to patients later during the day for completion at home. Electronic versions of the OES were equally sent a second time to some patients. Reminders and prompts were sent in the same way. Thirty-two patients completed and returned the second form for test-retest reliability.

### Instruments

#### The quick dash

The Disabilities of the Arm, Shoulder, and Hand (DASH) questionnaire is a PROM comprising 30 items developed to evaluate physical function and symptoms in patients with upper limb musculoskeletal disorders. It is a license-free PROM with a validated and reliable Chinese version (http://www.dash.iwh.on.ca/available-translations). The Quick Dash is a simplified version of the PROM comprising 11 items each with five options scored 1–5 and the optional high-performance sport/music or work modules (four items, scored 1–5). As part of this study, a smart device compatible version was designed for patient completion.

#### Short Form-36

The SF-36 is a generic health status PROM comprising 36 items over eight scale profiles. This can be classified under two headings: physical component summary (PCS) including physical functioning (PF), role physical (RP), bodily pain (BP), and general health (GH); mental component summary (MCS) including vitality (VT), social functioning (SF), role emotional (RE), and mental health (MH). The validated Chinese version was used [13] and as part of this study, a smart device compatible version was designed for patient use.

### Psychometrics

#### Internal consistency

The internal consistency of the questionnaire domains was assessed by calculating Cronbach’s α coefficients. Values of α in the range 0.80 to 0.90 are considered optimal, with a minimum α of 0.70 necessary to claim internal consistency [14].

#### Test-retest reliability (repeatability) and measurement error

Test-retest reliability was assessed with intraclass correlation coefficients by comparing Oxford elbow score domain scores obtained at the first outpatient visit with those completed at home more than 24 h later. To verify systemic change, the OES mean scores at test and retest sessions were compared using the paired *t* test. ICC ≥ 0.70 is adequate for patients enrolled in a clinical trial [14]. There are several parameters of measurement error including the standard error of measurement(SEM) which indicates measurement precision outcome with repeated measures and can be computed based on the ICC from the study population by the formula SEM = SD _pooled_ √1-ICC; Limits of agreement as proposed by Bland and Altman [15] which can be written as *d̄* ± 1.96 × √2 × SEM_consistency_ where *d̄* is the mean difference; and the coefficient of variation which is used to indicate reliability of apparatus in the phase of testing and calibration [16].

### Construct validity

To test the construct validity of the Oxford elbow score, Spearman’s correlation coefficients were calculated between the OES 3 domain subsets, the DASH and SF-36. According to studies from Juniper et al., correlation values of > 0.50, 0.35 to 0.50, and < 0.35 can be interpreted as strong, moderate, and weak, respectively [17]. Based on this and previous studies on OES construct validity [10, 18], we proposed the following hypothesis for convergent and discriminant validity.

1. Strong correlation coefficients (*r* > 0.5) between OES and the Quick Dash.
2. Moderate to strong correlations with related PCS domain scores of the SF-36: physical functioning (PF), role physical (RP), bodily pain (BP); and weak correlations with unrelated domain scores: general health (GH); mental component summary (MCS) including vitality (VT), social functioning (SF), role emotional (RE) and mental health (MH).

### Factor analysis

A confirmatory factor analysis was performed to evaluate the 3-factor structure of the OES in this new data set. The three factors (latent traits/unobserved factors) and their respective observed indicators (items) are as follows: Function—items 1,2,3,4; Pain—items 7,8,11,12; Social psychological—items 5,6,9,10. First, the Kaiser-Meyer-Olkin Measure of Sampling Adequacy (KMO) test and Bartlett’s Test of Sphericity were performed to assess the adequacy of the sample size for factor analysis calculation. Goodness of fit was then analyzed based on the factor loading, chi-square significance levels, relative χ^2^ (ratio of chi-square to degrees of freedom (χ^2^/df), goodness of fit index (GFI), adjusted goodness of fit index (AGFI), comparative fit index (CFI), non-normed fit index (NNFI), root mean square error of approximation (RMSEA), and standard root mean square residual (SRMR) [19]. Calculation estimates were carried out using IBM SPSS AMOS 26, and values were compared to their thresholds.

## Results

There were no missing items on completion of the forms; there was no ceiling effect (patients reporting the best possible score) or floor effect (patients reporting the worst possible score) for any of the 3 domains.

Thirty-two patients returned a completed OES a second time at least 24 h after first questionnaire completion, with an average time difference of 3.1 (SD 1.9) days from the first completion. The paired *t* test revealed no statistical significance (mean difference 0.438 , standard deviation 5.430, *p* > 0.05) between mean difference scores of the test and retest sessions implying that there was no significant systematic change between the intervals. Paired samples correlations showed strong correlations between the two sessions (*r* = 0.764) indicating patients maintained the same scoring range between the 2 sessions. The test-retest reliability calculated with ICC (consistency) was 0.764 and for the three different domains Function, Pain, and Social-psychological was 0.764, 0.624, and 0.590 respectively (Table 2). The Cronbach’s *α* coefficient was 0.906 and for the 3 different domains Function, Pain, and Social-psychological was 0.806, 0.796, and 0.776 respectively (Tables 3 and 4).

**Table 2:** Test and retest reproducibility determined by ICC_consistency_

OES domains	ICC	95% CI	*p* value
Function	.764	.570–.877	< 0.001
Pain	.624	.357–.797	< 0.001
Social-psychological	.590	.308–.776	< 0.001
Total	.764	.570–.877	< 0.001

*ICC* intraclass correlation, *CI* confidence interval

**Table 3:** Internal consistency as determined by Cronbach’s alpha for each domain

OES domains	Items	Cronbach’s α
Function	4	.806
Pain	4	.796
Social-psychological	4	.776
Total	12	.902

**Table 4:** Internal consistency for individual items on questionnaire

Item name and number	Mean (SD)	Corrected item-total correlation	Cronbach’s alpha item deleted
1.Difficulty with lifting	2.71 (.84)	.663	.893
2.Difficulty carrying bags	2.84 (.973)	.621	.895
3.Difficulty washing all over	3.01 (.93)	.581	.897
4. Difficulty dressing	3.21 (.70)	.623	.895
5. Elbow problem “controlling your life”	2.34 (1.13)	.710	.890
6. Elbow problem “on your mind”	2.47 (.99)	.654	.893
7. Pain in Bed at Night	2.64 (1.14)	.576	.899
8.Pain interfered with sleeping	3.19 (.89)	.565	.897
9.Interfered with usual work/everyday activities	2.73 (.78)	.673	.893
10.Limited leisure activities	2.76 (1.04)	.492	.902
11.Worst pain	2.50 (.760)	.755	.890
12.Usual pain	2.66 (.700)	.762	.890

*SD* standard deviation; table item reference [10]

The correlation coefficient between the QuickDASH and OES domains Function, Pain, and Social-psychological showed strong correlation (*r* > 0.5), *p* < 0.01. There were moderate correlations between OES domains and the physical functioning, role physical, and strong correlations with bodily pain subscales of the PCS domain of the SF-36; results were non-significant for all other subscales (Table 5).

**Table 5:** Correlation between the 3 domains of the OES, the Quick DASH, and PCS and MCS subscales of the SF-36

	OES			
	Total	Function	Pain	Social-psychological
SF-36				
PCS				
Physical functioning	.435**	.407*	.422**	.323
Role physical	.475**	.311	.442**	.534**
Bodily pain	.621**	.580**	.650**	.527**
General health	.256	.252	.315	.185
MCS				
Vitality	.082	.079	.179	− .015
Social functioning	.102	.065	.188	.151
Role emotional	.198	.076	.195	.237
Mental health	.070	− .020	.165	.050
QuickDASH	− .805**	− .824**	− .734**	− .622**

** Correlation is significant at the 0.01 level (two-tailed)

* Correlation is significant at the 0.05 level (two-tailed)

*PCS* physical condition scale, *MCS* mental condition scale

**Fig. 1:**
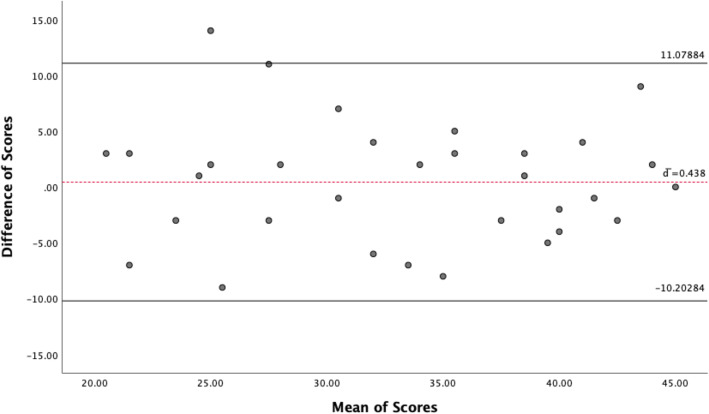
Bland and Altman plot—limits of agreement

Using the ICC (0.764) from the sample size, the SEM was 3.8. With 95% confidence interval, the limits of agreement were − 10.20284 (lower limit) and 11.07884 (upper limit). The Bland and Altman plot is depicted in Fig. 1.

**Fig. 2:**
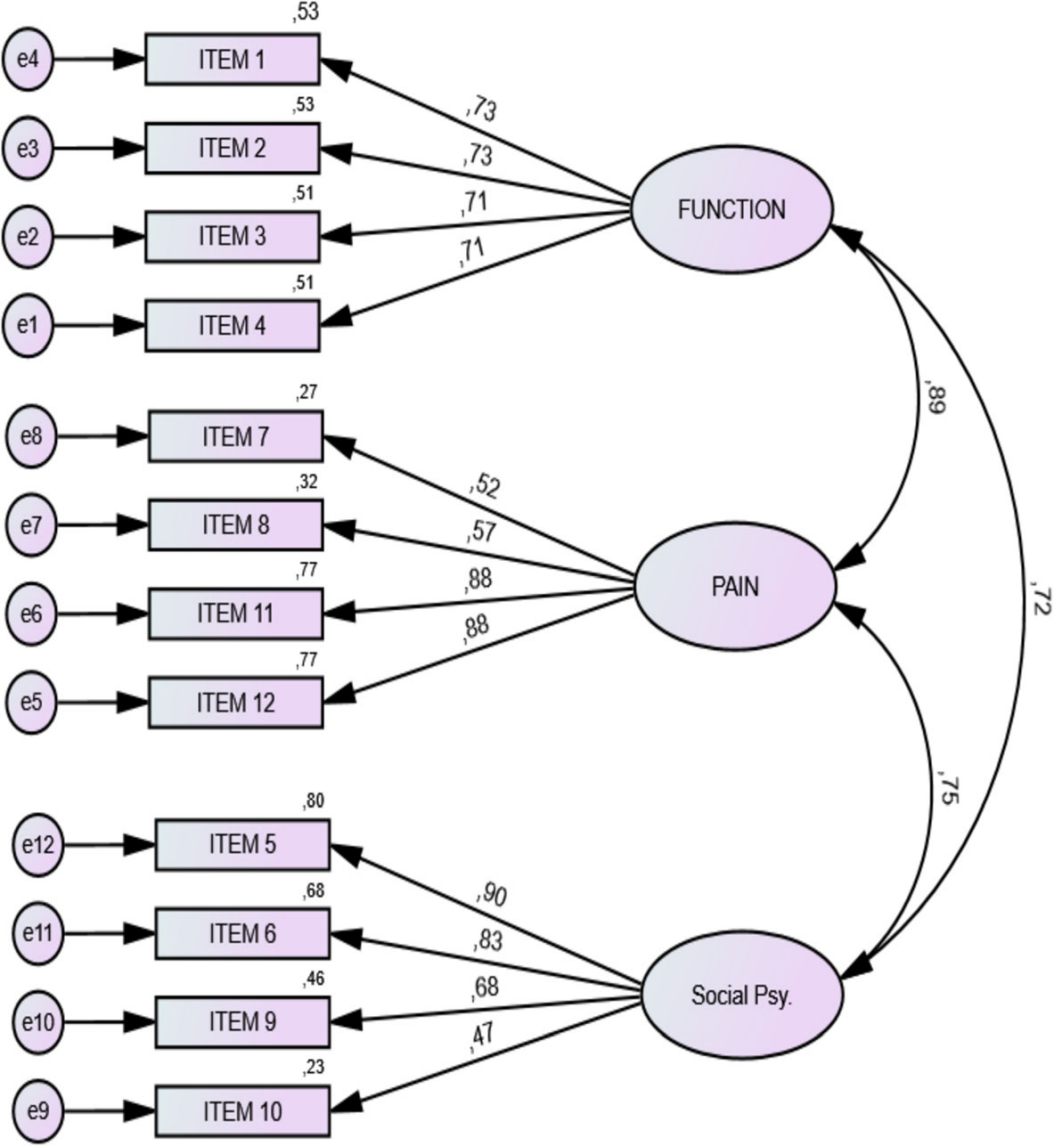
Confirmatory factor analysis—standardized factor loading between items and different OES domains. *PSY* psychological

KMO test revealed a value of 0.859, with values between 0.8 and 0.9 considered suitable [20]; and the Bartlett’s Test was significant at *p* < 0.0001, thus the sample was adequate for further analysis. Standardized estimates showing relationship between the latent and observed components, loading factor, and measurement error are illustrated in Fig. 2. The chi-square was 106.645, the degree of freedom was 51, and the deduced χ^2^/df was 2.09. Estimated values for indices of fit are as follows: goodness of fit index (GFI) 0.801, adjusted goodness of fit index( AGFI) 0.695, comparative fit index (CFI) 0.872, non-normed fit index (NNFI) 0.835, root mean square error of approximation (RMSEA) 0.126, and standard root mean square residual (SRMR) 0.091.

## Discussion

Findings from the study showed that the translated Chinese mandarin OES version (mainland) was reliable and valid. Reliability was measured using both the Cronbach’s α for internal consistency and the intraclass correlation. Results were classified as excellent and met the minimum recommended criteria of > 0.70 [14]. An overall measure of 0.902 was obtained for Cronbach α, and measures for the individual domains were less the 0.902 discarding any redundancy. These results were similar to results from the original OES study with overall Cronbach α measure of 0.9 and 0.90, 0.89, and 0.84, for Function, Pain, and Socio-psychological domains respectively [9].

The Chinese OES is equally reproduceable as confirmed by the overall test-retest reliability measure of 0.764, also meeting the minimum recommended criteria of ICC ≥ 0.70 [14]; ICC values for the Pain and Social-psychological domains fall short of the threshold but the overall ICC value is acceptable.

Similar studies by de Haan et al. on the validation of the Dutch OES version showed Cronbach’s α coefficient for the Function, Pain, and Social-psychological domains were 0.90, 0.87, and 0.90, respectively; intraclass correlation coefficients were 0.87, 0.89, and 0.87 respectively [21]. Studies by Ebrahimzadeh et al. showed that the overall ICC was 0.85 and 0.90, 0.76, and 0.75 for Function, Pain, and Social-psychological subscales, respectively. Cronbach’s alpha for Function, Pain, and Social-psychological subscales was 0.95, 0.86, and 0.85, respectively in the study [22].

Validity studies were assessed using Spearman’s correlation between Chinese OES domains and the QuickDASH evaluating similar aspects, and the SF-36. We hypothesized strong correlation between the OES and the QuickDASH score as well as similar domains from the physical component section of the SF-36. Results confirmed this hypothesis showing a strong correlation (*r* > 0.5) with the quick dash; 0.805 overall and for the three domains Function, Pain, and Social-psychological measures were − 0.824, − 0.734, and − 0.622 respectively. This study showed moderate correlation with the physical functioning, role physical subscales of the PCS, 0.435 and 0.475 respectively; and strong correlations with bodily pain 0.621. Results from the general health subscale of the PCS and all MCS subscales were non-significant. Studies by Yosmaoglu et al. showed non-significant results for correlation between the general health and vitality subscales [18]. The original OES study showed divergent validity with low correlations between all three Oxford elbow score domains and the SF-36 mental health and general health perception domains [9].

The chi-square (χ2) value was significant at *p* < 0.05 implying an inadequate fit. But chi-square values vary with sample sizes so single χ^2^ results cannot be used to determine goodness of fit. The value of relative χ^2^ fell within the threshold of ≤ 2.5, so it can be interpreted as an excellent fit. However, other indices for fit evaluation fell short of the threshold, and none of the 2-index presentation strategy by Hu and Bentler’s fulfilled excellent fit criteria [23]. Notwithstanding, the standardized factor loadings were acceptable indicating adequate correlation of the items to their respective constructs. Research by Yosmaoglu et al. supported the 3-factor structure with an excellent relative chi-square value, and acceptable threshold for all other parameters except AGFI which had a lower value and RMSEA with a high value [18].

SEM and Bland Altman plot with the limits of agreement are important parameters for evaluation of responsiveness and interpretability. Limits of agreement give an indication of the variation of scores in a stable patient. From these, we can compute the smallest detectable change (SDC), also known as minimal detectable change (MDC) as well as the minimal important change (MIC). The SDC can be calculated as 1.96 × √2 × SEM, which is 11 points in this study. So, following longitudinal studies with changes in patients score, the clinician can be able to interprete if changes are either due to measurement error for changes in the range of the limits of agreement or below the SDC or real clinical change for values greater than the MIC cut off value. Values from this study can be used in other studies with the same sample population to further evaluate responsiveness and interpretability.

This study made use of electronic PROMs instead of the traditional paper-based PROMs for collection of data. Previous studies have investigated advantages of using e-PROMs and advocate their use to increase efficiency of work and resources [24]. Questionnaires in our study were sent to patients via the ubiquitous social media WeChat® platform. Overall, this was well tolerated by the patients who consented to take part in the study. Further studies on the efficiency of different PROM collection methods have to be carried out to ascertain suitable PROM collection protocol.

This study had some limitations; first, the sample size of 32 which was used for ICC and limits of agreement was relatively small, and the sample used for this study was representative of one Mandarin speaking city. Intepretability and responsiveness were not addressed in this study. Future longitudinal studies should be carried out to asses these two measurement properties and other variants of Chinese language including traditional Chinese should be equally used for the PROM to address a wider population, as well as studies on effective methods of PROM collection.

## Conclusion

The Chinese mandarin OES is reliable and valid 12 item score that can be used in the evaluation of patients with elbow disorders in the Chinese population.

## Data Availability

The datasets generated during the current study are not publicly available due to the fact that some data sets contain participant personal information such as names and phone numbers.

## Abbreviations

OES: Oxford Elbow score
ICC: Intraclass correlation coefficient
CFA: Confirmatory factor analysis
PROMs: Patient-reported outcome measures
COSMIN: Consensus-Based Standards for the Selection of Health Measurement Instruments
QuickDASH: Quick Disabilities of the Arm Shoulder and Hand score
PRTEE: Patient-Rated Tennis Elbow Evaluation
PCS: Physical component summary
PF: Physical functioning
RP: Role physical
BP: Bodily pain
GH: General health
MCS: Mental component summary
VT: Vitality
SF: Social functioning
RE: Role emotional
MH: Mental health
SEM: Standard error of measurement
KMO: Kaiser-Meyer-Olkin Measure of Sampling Adequacy
GFI: Goodness of fit index
AGFI: Adjusted goodness of fit index
CFI: Comparative fit index
NNFI: Non-normed fit index
RMSEA: Root mean square error of approximation
SRMR: Standard root mean square residual
SDC: Smallest detectable change
MDC: Minimal detectable change
MIC: Minimal important change
